# On the Antecedents of an Electrophysiological Signature of Retrieval Mode

**DOI:** 10.1371/journal.pone.0167574

**Published:** 2016-12-09

**Authors:** Angharad N. Williams, Lisa H. Evans, Jane E. Herron, Edward L. Wilding

**Affiliations:** 1 Cardiff University Brain Research Imaging Centre (CUBRIC), School of Psychology, Cardiff University, Cardiff, United Kingdom; 2 Institute of Psychological Medicine and Clinical Neurosciences (IPMCN), School of Medicine, Cardiff University, Cardiff, United Kingdom; 3 MRC Centre for Neuropsychiatric Genetics and Genomics, School of Medicine, Cardiff University, Cardiff, United Kingdom; 4 School of Psychology, Nottingham University, Nottingham, United Kingdom; University of Akron, UNITED STATES

## Abstract

It has been proposed that people employ a common set of sustained operations (retrieval mode) when preparing to remember different kinds of episodic information. In two experiments, however, there was no evidence for the pattern of brain activity commonly assumed to index these operations. In both experiments event-related potentials (ERPs) were recorded time-locked to alternating preparatory cues signalling that participants should prepare for different retrieval tasks. One cue signalled episodic retrieval: remember the location where the object was presented in a prior study phase. The other signalled semantic retrieval: identify the location where the object is most commonly found (Experiment 1) or identify the typical size of the object (Experiment 2). In both experiments, only two trials of the same task were completed in succession. This enabled ERP contrasts between ‘repeat’ trials (the cue on the preceding trial signalled the same retrieval task), and ‘switch’ trials (the cue differed from the preceding trial). There were differences between the ERPs elicited by the preparatory task cues in Experiment 1 only: these were evident only on switch trials and comprised more positive-going activity over right-frontal scalp for the semantic than for the episodic task. These findings diverge from previous outcomes where the activity differentiating cues signalling preparation for episodic or semantic retrieval has been restricted to right-frontal scalp sites, comprising more positive-going activity for the episodic than for the semantic task. While these findings are consistent with the view that there is not a common set of operations engaged when people prepare to remember different kinds of episodic information, an alternative account is offered here, which is that these outcomes are a consequence of structural and temporal components of the experiment designs.

## Introduction

Tulving [1] defined retrieval mode as a cognitive set, entry to which ensures that subsequent experiences will be treated as cues for retrieval of information from episodic memory. When he introduced retrieval mode he described it as a generic set that will be engaged irrespective of what kind of episodic content people might prepare to recover [2], and he also commented on what he saw as difficulties in testing for its existence and properties. This was perhaps a slightly narrow perspective, in so far as task-switching manipulations and real-time measures of neural activity were tools that were then available, and could be employed either jointly or in isolation to investigate cognitive states and their sequelae. It was none the less the case that interest in the concept of retrieval mode, its neural basis and its functional properties was developed substantively in the early- to mid-nineties when Positron Emission Tomography (PET) was employed in studies of human memory [3–9].

Research into the temporal and functional properties of retrieval mode was then taken forward in several electrophysiological studies. Düzel and colleagues [10, 11] acquired direct current (DC) potentials while participants alternated between completing recognition memory and semantic memory tasks. Participants completed four trials of each task before being cued to switch to the alternate task, and ERPs were acquired time-locked to the cues signalling which task to complete. Relative to the activity elicited in response to the semantic cues, the activity elicited by cues for the episodic (recognition memory) task was more positive-going at right-frontal scalp sites. This divergence emerged soon after the task-cues and was sustained across the four-trial sequence. Düzel and colleagues proposed that this temporally extended modulation was a neural signature of retrieval mode [10], and in a subsequent PET study suggested that the neural generators responsible for the signature were in right pre-frontal cortex (PFC) [11].

Morcom and Rugg [12] employed the same task pair as Düzel, Cabeza [10]. However, they cued participants on each trial as to which task to complete and switches between tasks were frequent. Morcom and Rugg [12] contrasted ERPs elicited by cues on the first trial after a switch from the alternate task (a switch trial) to trials where the same task had been completed on the immediately preceding trial (a repeat trial). Differences according to cue-type were reliable on repeat trials only. They comprised a greater relative positivity over right-central and -frontal scalp elicited by episodic in comparison to semantic task cues. They suggested that this activity was linked to retrieval mode, and argued that the presence of the signature on repeat trials reflected the fact that adopting retrieval mode was not something that occurred spontaneously.

In an important further study, Herron and Wilding [13] again employed a task-switching design and in a departure from earlier work asked participants to switch between three tasks. One of these three was the semantic task used by Düzel, Cabeza [10] and by Morcom and Rugg [12]. The other two required episodic retrieval: of either the spatial location in which study words had been shown or the encoding task completed on study words. The critical finding was that a temporally extended greater relative positivity was observed on both episodic tasks relative to the semantic task, and this was reliable on repeat trials only. This was the first within-experiment demonstration that this neural signature did not differentiate between the episodic content that people were preparing to recover.

The same pattern of effects across switch and repeat trials has been observed in several subsequent studies, typically accompanied by reaction time switch costs; slower reaction times on switch than on repeat trials [13, 14]. In one recent notable exception, however, Evans, Williams [15] observed a temporally sustained right-frontal modulation associated with preparation for episodic retrieval on switch rather than on repeat trials. In that experiment, the episodic task required a judgment about the location (left- or right-hand side of screen) in which items had been shown in a prior study phase. The alternate task also required a location judgment. In this case it was the position of the test stimulus on the screen.

Evans, Williams [15] chose this task pairing in order to address a concern that arose from an assessment of the circumstances under which putative ERP indices of retrieval mode had been observed. They noted that within the tasks requiring episodic or semantic retrieval the information content required to make a judgment also varied. For example, Düzel, Cabeza [10] and Morcom and Rugg [12] both required old/new recognition and judgments about whether items denoted by test words were best described as animate or inanimate. Herron and Wilding [13, 14] contrasted episodic judgments about which encoding task had been completed and/or episodic location judgments with semantic judgments about whether the referents of test words could move of their own accord.

Evans, Williams [15] observed that this confound between memory type (episodic/semantic) and the kinds of contents that were required for test judgments raised the possibility that the effects reported previously might reasonably be attributed to the challenges posed by recovery of distinct kinds of contents (and the subsequent decisions that were required) rather than to a general imperative to prepare for episodic retrieval. This observation motivated their choice of two spatially oriented tasks: location judgments in their episodic task and a related perceptual judgment task. The fact that they observed a morphologically similar effect to that obtained in the preceding studies prompted their claim that the right-frontal modulation is an index of retrieval mode. In support of this account, they also demonstrated that the differences between preparatory activities on switch and repeat trials were carried only by the activities for the episodic task [for a consistent outcome, see: 14]. Evans, Williams [15] also suggested that the putative index of retrieval mode occurred on switch trials in their experiment because of the requirement to focus on spatial information in both tasks. They argued that this degree of similarity afforded a quicker transition into retrieval mode than had been possible in previous studies where the kinds of contents to which judgments were required were somewhat more distinct.

While this account may well be correct, the degree of similarity between contents is not the only difference between the design employed by Evans, Williams [15] and those used in previous studies. Most notable is the use of a perceptual task in the study by Evans, Williams [15] whereas a task requiring semantic retrieval was paired alongside one or more episodic tasks in previous studies. This further difference raises the possibility that the presence of a putative index of retrieval mode on switch trials is due to this change across tasks rather than the content similarity manipulation.

The first experiment reported below was designed in order to permit an assessment of the proposal made by Evans, Williams [15] for the presence of a putative index of retrieval mode on switch trials. If their account regarding content is correct, then the same results should be obtained irrespective of the kind of task in which attention to content is encouraged. Evans, Williams [15] contrasted neural activity obtained in a task requiring episodic judgments with one requiring perceptual judgments. In this experiment the contrast is between tasks requiring episodic or semantic judgments. Critically, in both cases the focus on spatial information remains. Towards this end, participants were initially shown objects either inside or outside the outline of a building. They were aware that their memory for the objects and their locations would be tested subsequently. At test participants alternated between making memory judgments to objects (inside/outside/new) with semantic judgments about common object locations (inside/outside/both). Failure to replicate the findings of Evans, Williams [15] in this design would challenge their view that a shared focus on spatial contents across tasks was responsible for the fact that they observed a putative index of retrieval mode on switch rather than on repeat trials.

## Method

### Participants

A sample size of 24 was decided *a priori* based on counterbalancing considerations and power analyses for a replication attempt of the effects found previously (e.g. Evans, Williams [15]: *d*_*z*_ = 0.55, α = 0.05, 1-β = 0.80, N = 22). Data were collected from 26 participants, as the data from two participants were excluded: one due to excessive EEG artefact and one due to a semantic categorisation score of below 40%. Demographics for the 24 participants were: mean age = 21, range = 18–26, 19 female. All participants gave written informed consent before participating, and were right-handed, with normal or corrected-to-normal vision. None of the participants had a diagnosis of dyslexia, and they were all native English speakers. At the time of testing no participants reported using psychotropic medication. Participants were paid £10 per hour and each testing session lasted a maximum of two hours. Cardiff University School of Psychology Ethics Committee reviewed and approved this research.

### Stimuli

These were 240 black line drawings of objects, selected from the International Picture Naming Project Database [16]. This included animate and inanimate stimuli, for example: animals, everyday household objects, tools, transport vehicles, food items, and jewellery. The corresponding name for each object was between three and ten letters in length, the percentage picture naming frequency was above 0.80, and the frequency range was between zero and 7.396 (CELEX log transformed). The objects were presented on a monitor with a white background, positioned one metre directly in front of participants. The stimuli subtended maximum visual angles of 5.4° vertically and 8.5° horizontally at study. At test, objects were presented in the centre of the screen subtending maximum visual angles of 1.6° vertically and 1.7° horizontally. Prior to the experiment the objects were classified into one of three semantic categories, according to where they were commonly found: inside, outside or were equally like to be found inside or outside. There were 80 objects in each semantic category, and for this classification the mean inter-rater reliability of three raters in a piloting session was 0.72.

### Design and procedure

The experiment had five study-test cycles, and the 80 stimuli from each semantic category (inside/outside/both) were allocated to one of five lists such that each 48-item list had an equal number (16) from each semantic category. A further 48 items were selected and used to form two additional practice blocks, each half the length of the other five study-test blocks. These were used to familiarise participants with the experiment demands.

**Fig 1:**
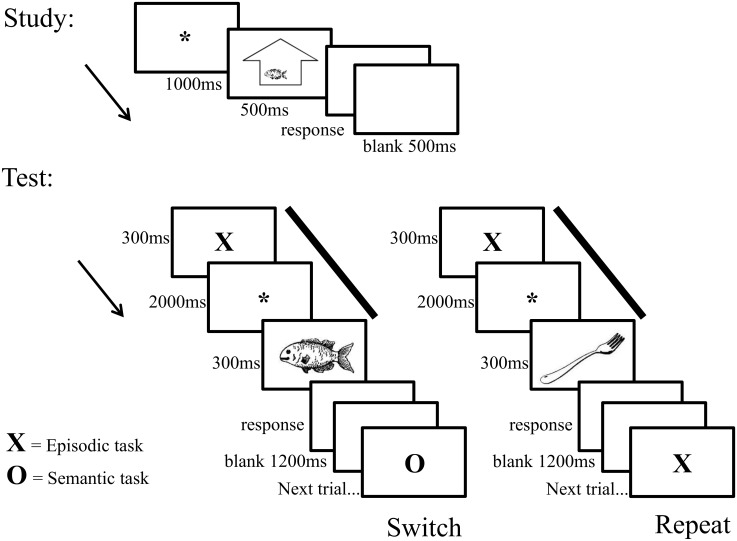
A schematic illustration of trial sequences at study (upper panel) and on switch and repeat trials at test (lower panel). At study, participants were shown an object either inside or outside of an abstract outline of a building (response options: inside/outside). At test, participants were shown either an X or O cue. Following the X cue, participants were required to prepare for the episodic task: to give the study location of the object which follows (response options: inside/outside/new). Following the O cue, in Experiment 1, participants were required to prepare for the semantic task: to give the usual location of the object which follows (response options: inside/outside/both). In addition, the solid bars indicate the preparatory period of interest.

In each study phase of each cycle, an equal number of objects (12) were shown inside or outside an abstract outline of a building in one of eight locations (see Fig 1). At the start of each trial an asterisk was presented in the middle of the screen for 1000 milliseconds (ms). This was followed by an object (presented inside or outside the building outline) for 500ms. The monitor was then blank until a response was made, and remained blank for a further 500ms before the next trial began. Participants were asked to indicate whether the object appeared inside or outside via button press with their middle or index fingers, respectively.

In each test phase the 24 objects from the immediately preceding study phase were presented intermixed with 24 unstudied objects. Each test object was preceded by one of two preparatory cues that were presented in the centre of the screen. These cues indicated which task participants were to prepare to complete. ‘X’ directed participants to prepare for the episodic task (response options: old inside/old outside/new). ‘O’ directed participants to prepare for the semantic task. This task required identification of the common location of the object depicted (response options: inside/outside/both). Each preparatory cue appeared for 300ms and was followed by a central asterisk for 2000ms (see Fig 1). An object was then shown in the centre of the screen for 300ms. The monitor was then blank until a response was made, and remained blank for a further 1200ms before the next trial began. Participants were asked to respond as quickly and as accurately as possible. Trials on which responses were faster than 300ms or slower than 4000ms were counted as errors and excluded from the behavioural analyses (0.9% of the trials).

Each cue-type was always presented for two consecutive trials—hereafter a switch and a repeat trial—and an equal number of studied and unstudied objects followed each cue-type. For objects, their presence on switch or repeat trials, the task they were encountered in (episodic or semantic), their old/new status, and their study location (inside/outside) were counterbalanced across participants. During the test phase, responses were made using the same fingers as at study, with the addition of the index finger of the other hand to indicate ‘new’ or ‘both’, for the episodic and the semantic tasks, respectively. The hands used for these responses were balanced across participants.

### Electroencephalogram (EEG) procedures

EEG was acquired continuously relative to an average reference via silver/silver chloride (Ag/AgCl) electrodes embedded in an electrode cap (bandpass filter = 0.03-40Hz, 24dB/octave; sampling rate = 250Hz) from 25 scalp sites at midline (Fz, Cz, Pz) and left/right hemisphere locations comprising fronto-polar (Fp1/Fp2), frontal (F7/F8, F5/F6, F3/F4), central (T7/T8, C5/C6, C3/C4), posterior (P7/P8, P5/P6, P3/P4), and occipital (O1/O2) sites. Additional electrodes were placed above and below the right eye, and on the outer canthi. Electrodes were also placed on the mastoid processes. Impedance at each electrode/scalp interface was below 5KΩ at the start of each recording session. ERPs elicited by the preparatory cues were segmented into epochs of 2500ms duration including a 200ms pre-stimulus baseline relative to which all post-stimulus voltages were computed. Eye blinks were corrected using the algorithm recommended by Gratton, Coles [17]. In addition, trials containing residual eye movement artefact or other outliers were rejected during visual inspection after completing automated detection and rejection of artefacts which were conducted as follows; maximum and minimum allowed amplitude (+/- 100μV), gradient voltage step per sampling point (75μV/ms), and low activity levels (0.5μV/50ms). The first trial in each test block was removed from analyses, as it is neither a switch nor a repeat trial. There were four conditions: ERPs elicited by the episodic and semantic preparatory cues on switch and on repeat trials. On average, 84% of the trials contributed to the ERP data for each participant. Mean trial numbers contributing to the ERPs (ranges in parentheses) were: episodic switch = 50 (34–58), episodic repeat = 52 (32–60), semantic switch = 49 (31–58), semantic repeat = 51 (31–60).

## Results

### Behaviour

During the study phases participants correctly responded ‘inside’ or ‘outside’ on 96% of trials. Table 1 shows the response accuracy data for the test phases. Discrimination scores (discrimination index: *Pr* = *p*(hit)–*p*(false alarm)) were calculated by collapsing across the accuracy of location (inside/outside) judgments for old objects [18]. In preliminary analyses there were no differences in accuracy according to the inside/outside dimension and data are shown collapsed across this dimension. They were above zero for both trial-types (switch Pr: 0.58, *t*(23) = 14.85, *p* < 0.001, *d*_*z*_ = 3.03, 99.8% CL; repeat Pr: 0.65, *t*(23) = 18.83, *p* < 0.001, *d*_*z*_ = 3.84, 99.9% CL (Common Language effect size statistic, [19, 20]) and higher on repeat than on switch trials (*t*(23) = 2.56, *p* < 0.05, *d*_*z*_ = 0.52, 70% CL). The probabilities of correct location judgments for items given a correct ‘old’ response and collapsed across the inside/outside dimension (see Table 1) were reliably above chance on switch (*t*(23) = 9.16, *p* < 0.001, *d*_*z*_ = 1.87, 97% CL) and repeat trials (*t*(23) = 13.33, *p* < 0.001, *d*_*z*_ = 2.72, 99.7% CL). Performance was superior on repeat trials (*t*(23) = 2.77, *p* < 0.05, *d*_*z*_ = 0.57, 71% CL). For the semantic task, the probability of classifying the item according to the modal rating given by the original raters was equivalent for switch and repeat trials (0.73).

**Table 1:** Experiment 1: Probabilities of correct old, new and location judgments in the episodic task and correct classifications in the semantic task on switch and repeat trials. Probabilities for Old words were calculated by collapsing across correct and incorrect location judgments. The Location values are the conditional probabilities of a correct inside or outside judgment. Standard deviations are in parentheses.

	Switch	Repeat
*Episodic*		
Old	0.85 (0.14)	0.87 (0.13)
New	0.73 (0.19)	0.78 (0.17)
Location	0.75 (0.13)	0.81 (0.11)
*Semantic*		
Classification	0.73 (0.08)	0.73 (0.07)

A 2x2x2 ANOVA was conducted on the mean Reaction Times (RTs) for correct responses (Table 2). For this analysis the RTs were separated by cue-type (episodic/semantic), trial-type (switch/repeat) and old/new status. For the episodic task the ‘correct’ old response is the probability of a correct location judgment (collapsed across the inside/outside dimension). Main effects of trial-type (*F*(1, 23) = 16.32, *p* = 0.001, *d*_*z*_ = 0.82, 80% CL), and status (*F*(1, 23) = 18.53, *p* < 0.001, *d*_*z*_ = 0.88, 81% CL), were moderated by a trial-type by status by cue-type interaction (*F*(1, 23) = 4.40, *p* < 0.05, *η*_*p*_^*2*^ = 0.16). There was also an interaction between cue-type and old/new status (*F*(1, 23) = 5.36, *p* < 0.05, *η*_*p*_^*2*^ = 0.19).

**Table 2:** Experiment 1: Mean reaction times (ms) for correct responses on each task on switch and repeat trials. Standard deviations are in parentheses.

	Switch	Repeat
*Episodic task*:		
Old	1509 (478)	1412 (381)
New	1327 (356)	1226 (287)
*Semantic task*:		
Old	1483 (368)	1342 (338)
New	1385 (313)	1367 (397)

The data were then analysed within each cue-type. For the episodic task responses were faster on repeat than switch trials (*F*(1, 23) = 6.81, *p* < 0.05, *d*_*z*_ = 0.53, 70% CL) and faster for old than for new objects (*F*(1, 23) = 11.70, *p* < 0.05, *d*_*z*_ = 0.70, 76% CL). For the semantic task a main effect of trial-type (*F*(1, 23) = 12.00, *p* < 0.05, *d*_*z*_ = 0.71, 76% CL) was moderated by an interaction between this factor and status (*F*(1, 23) = 6.30, *p* < 0.05, *η*_*p*_^*2*^ = 0.22). The RT advantage for repeat over switch trials is markedly larger for old than for new objects.

### ERP analyses

**Fig 2:**
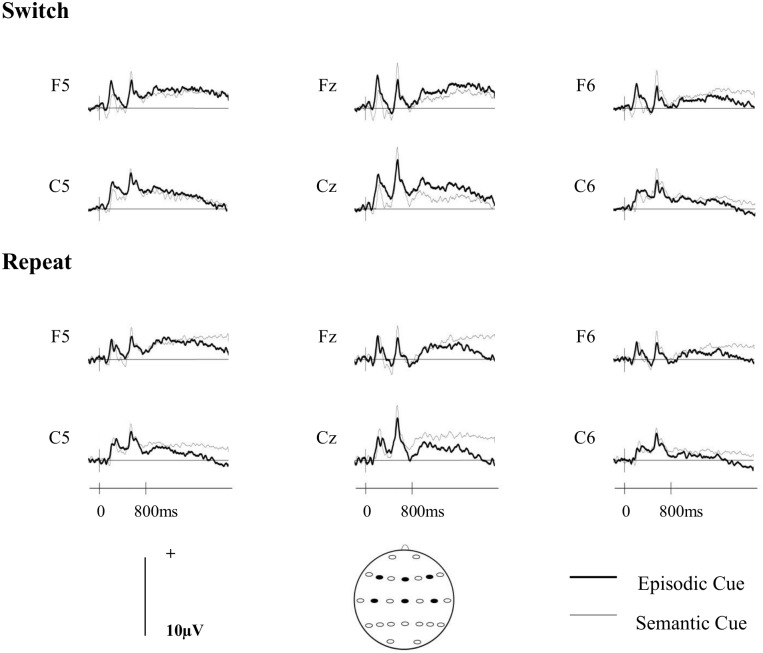
Grand averaged ERPs separated according to cue-type (episodic/semantic) on switch (upper panel) and repeat (lower panel) trials for midline (Fz, Cz) and left- and right- frontal (F5, F6) and central (C5, C6) electrode locations.

**Fig 3:**
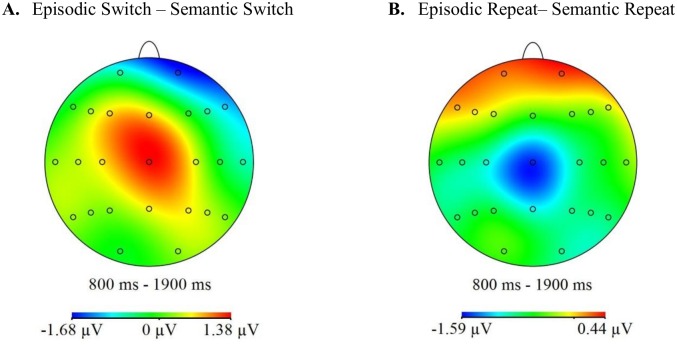
Topographic maps calculated using a spherical spline interpolation. The maps were computed from difference scores obtained by subtracting mean amplitudes associated with semantic cues from those associated with the episodic cues on switch (A) and repeat (B) trials for the 800 to 1900ms time window. The scale below each map denotes the voltage range (μV) of the differences between cue-elicited activities.

Fig 2 shows the grand averaged ERP waveforms for each cue- and trial-type at midline, left and right anterior and central sites. Topographic maps depicting the differences between the scalp distributions of the ERPs associated with the two cue- and trial-types are shown in Fig 3. On switch trials there is a small greater relative positivity at right-frontal sites for the semantic relative to the episodic cues, which is reversed at central sites. On repeat trials there is a greater relative positivity for the semantic relative to the episodic cues at central locations.

The analysis strategy followed closely that employed by Evans, Williams [15]. The analyses were conducted on mean amplitudes taken over the 800 to 1900ms post-stimulus time window, and following similar approaches in other studies [12–14] the initial analysis included 12 sites distributed over left- and right-hemisphere frontal and central scalp (F3/F4, F5/F6, F7/F8, C3/C4, C5/C6, T7/T8) within an ANOVA incorporating the factors of cue-type (episodic/semantic), trial-type (switch/repeat), location in the anterior-posterior plane (anterior/central), hemisphere (left/right), and site (inferior/mid-lateral/superior). Only outcomes involving the factor of cue-type are reported.

The initial analysis revealed an interaction between all five factors (*F*(1.7, 39.3) = 4.35, *p* < 0.05, *η*_*p*_^*2*^ = 0.16). There were also lower order interactions involving: cue-type and hemisphere (*F*(1, 23) = 4.62, *p* < 0.05, *η*_*p*_^*2*^ = 0.17), cue-type, trial-type, and the anterior-central dimension (*F*(1, 23) = 4.37, *p* < 0.05, *η*_*p*_^*2*^ = 0.16) and cue-type, the anterior-central dimension, and hemisphere (*F*(1, 23) = 6.70, *p* < 0.05, *η*_*p*_^*2*^ = 0.23).

In light of this outcome, separate ANOVAs were carried out for switch and repeat trials, and in both cases reliable differences according to cue-type were evident. For switch trials the analyses revealed interactions between: cue-type, anterior-central, hemisphere and site (*F*(1.7, 39.4) = 4.66, *p* < 0.05, *η*_*p*_^*2*^ = 0.17), and cue-type and hemisphere (*F*(1, 23) = 8.41, *p* < 0.05, *η*_*p*_^*2*^ = 0.27). Follow up ANOVAs were subsequently carried out separately for the anterior and central sites and revealed reliable differences at anterior sites only. An interaction between cue-type and hemisphere (*F*(1, 23) = 8.31, *p* < 0.05, *η*_*p*_^*2*^ = 0.27) reflects the right-lateralisation of the greater relative positivity for ERPs elicited by the semantic rather than the episodic cues. An interaction between cue-type and site (*F*(1.3, 29.6) = 4.00, *p* < 0.05, *η*_*p*_^*2*^ = 0.15) reflects the fact that this relative positivity is largest at inferior scalp locations.

The analysis on repeat trials revealed an interaction between cue-type and the anterior-central dimension (*F*(1, 23) = 4.31, *p* < 0.05, *η*_*p*_^*2*^ = 0.16). Separate follow up analyses at anterior and central sites revealed no reliable outcomes and the interaction term reflects primarily the greater relative positivity for the semantic than for the episodic cues at the vertex.

### Bayesian ERP analyses

**Fig 4:**
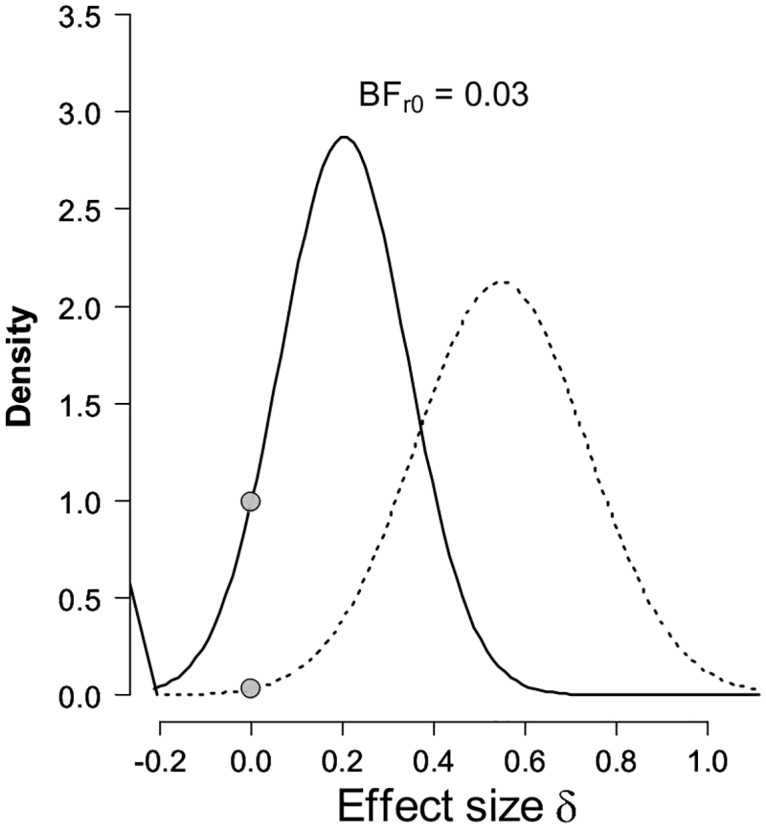
Bayesian results of the replication test for the right-frontal positivity identified previously during preparation for episodic memory retrieval on switch trials. The dotted line represents the posterior from the original study [15], which was used as the prior for the effect size in the replication test. The solid line represents the posterior distribution after the data from the replication attempt (Experiment 1) are taken into account. The grey dots indicate the ordinates of this prior and posterior for the null hypothesis that the effect size is zero. The ratio of these two ordinates gives the result of the replication test [22].

Bayes Factors (BFs) were also calculated in order to investigate the strength of evidence for either the null (no index of retrieval mode) or alternative hypothesis [21, 22]. BFs 3.0 and greater were considered as substantial evidence for the alternative hypothesis, whereas values 0.33 and below were considered substantial evidence for the null [23, 24]. BFs were calculated using the R-version of the Replication Test [22]. The t-statistics and sample sizes for this experiment were contrasted with those from the data acquired by Evans, Williams [15]. The t-statistic is the value obtained for the contrast between mean amplitudes on switch trials for the two trial-types. The data entering this contrast were mean amplitude measures averaged across three right-frontal electrode locations (F4, F6, F8) for the 800-1900ms post-stimulus epoch. For this experiment, sample size (N) = 24, t = -1.03; for Evans, Williams [15], N = 32, t = 3.09. When considered as a replication of the divergence obtained on switch trials in Evans, Williams [15] the BF = 0.03 (Fig 4), providing very strong evidence in favour of the null [23, 24].

## Discussion

This experiment was designed to test the proposal that a greater relative positivity at right-frontal scalp sites on switch trials would emerge in designs where participants switched between episodic and non-episodic tasks in which the kinds of information to be processed were similar. This prediction was based on the findings and subsequent interpretation offered by Evans, Williams [15] in an experiment where participants switched between a retrieval task requiring judgments about study location, and a task requiring judgments about where on screen test words were shown. ERPs elicited by cues signalling which task to prepare to complete diverged only on switch trials, where those associated with the episodic cue were more positive-going over right-frontal scalp. In the experiment reported here judgments about location were again required in both tasks. In the episodic task, the location manipulation was whether objects had appeared inside or outside a representation of a building in a prior study phase. In the semantic task the requirement was to denote the location in which objects were typically found.

The findings of Evans, Williams [15] on switch trials were not replicated, and perhaps more surprisingly, there was no evidence for a greater relative positivity in ERPs elicited by episodic cues on repeat trials either. The ERP outcomes in this experiment therefore converge with few existing data points. The pattern of behavioural data is broadly consistent with previous findings, in so far as switch costs were observed, but further comment on these data is deferred until the outcomes of a second experiment are reported. This second experiment also required participants to switch between two tasks, and one of these was the episodic task employed in the experiment already described. The other task required semantic judgments, but rather than requiring location judgments it required a size judgment. The intention here was to assess the correlates of preparation for retrieval in a design where the kinds of information required for the task judgments diverge, which is a design comparable to that used in the vast majority of previous studies in which a putative signature of retrieval mode has been observed on repeat trials [12–14, 25].

## Experiment 2: Introduction

This experiment was conducted with a view to understanding the failure to replicate previous findings in Experiment 1. A strong interpretation of the findings in Experiment 1 is that, contrary to previous claims, there is not a generic index of retrieval mode that is observed during preparation for episodic retrieval. This view is perhaps premature. Experiment 2 was designed to explore correlates of preparatory retrieval processing in the episodic task used in Experiment 1 contrasted with a second task used in the majority of prior studies: one that requires semantic memory judgments for a kind of content that differs substantively from that required in the episodic task.

## Methods

All elements were the same as Experiment 1 with the following exceptions. The substantive change was the replacement of the semantic location task with a semantic size judgment task. The changes to the design and procedure as well as other numerical differences across the experiments are described below. EEG acquisition procedures were identical.

### Participants

Data were collected and analysed from 32 people (mean age = 22, range = 18–28, 21 female). This sample size was determined via the Bayesian Stopping Rule [21] with a first look conducted at 24 participants: the number of participants in the first experiment in this report.

### Design and procedure

Numbers of objects and the resources they were taken from were the same as Experiment 1. In this experiment, however, the objects were classified into one of three semantic categories according to the size of the object depicted: smaller than a lunchbox, larger than a lunchbox but smaller than a suitcase, larger than a suitcase. There were approximately a third of the objects in each semantic category (88, 62, and 90, respectively). For the semantic classification, the mean inter-rater reliability of three raters was 0.66. The experiment comprised five study-test cycles, and for each cycle each of the stimuli from each semantic category were randomly assigned to one of five lists. Thus, each list contained 48 objects: 17/18 small, 12/13 medium, and 18 large objects. Timings and trial sequences were as for Experiment 1 (see Fig 1).

All test elements matched Experiment 1, with the exception that the ‘O’ cue at test signalled preparation for a size judgment (<lunchbox, >lunchbox<suitcase, >both). 0.6% of trials were rejected because responses were faster than 300ms or slower than 4000ms.

On average, 86% of the switch and repeat trials contributed to the ERP cue data for each participant. The mean trial numbers contributing to the ERPs (ranges in parentheses) were: episodic switch = 50 (27–57), episodic repeat = 53 (31–60), semantic switch = 51 (34–58), semantic repeat = 52 (29–59).

## Results

### Behaviour

During the study phases participants correctly responded ‘inside’ or ‘outside’ on 98% of trials. Table 3 shows the response accuracy data for the test phases. Discrimination scores (discrimination index: *Pr* = *p*(hit)–*p*(false alarm)) for the episodic task were above zero for both trial-types (switch Pr: 0.61, *t*(31) = 17.74, *p* < 0.001, *d*_*z*_ = 3.14, 99.9% CL; repeat Pr: 0.68, *t*(31) = 16.94, *p* < 0.001, *d*_*z*_ = 2.99, 99.8% CL) and higher on repeat trials than on switch trials (*t*(31) = 2.95, *p* < 0.05, *d*_*z*_ = 0.52, 70% CL). The conditional probabilities of correct source judgments collapsed across the inside/outside dimension (see Table 1) were reliably above chance in both cases (switch: *t*(31) = 11.70, *p* < 0.001, *d*_*z*_ = 2.07, 98% CL; repeat: *t*(31) = 13.31, *p* < 0.001, *d*_*z*_ = 2.35, 99% CL). For the semantic task, the probability of classifying the item according to the modal rating given by the original raters was equivalent for switch and repeat trials (0.75).

**Table 3:** Experiment 2: Probabilities of correct old, new and location judgments in the episodic task and correct classifications in the semantic task on switch and repeat trials. All other details as for Table 1.

	Switch	Repeat
*Episodic task*:		
Old	0.86 (0.10)	0.85 (0.12)
New	0.75 (0.19)	0.83 (0.17)
Location	0.76 (0.13)	0.77 (0.11)
*Semantic task*:		
Classification	0.75 (0.08)	0.75 (0.08)

The 2x2x2 ANOVA on mean RTs (Table 4) with factors of cue-type (episodic/semantic), trial-type (switch/repeat), and status (old/new) revealed several effects. Main effects of trial-type (*F*(1, 31) = 11.97, *p* < 0.05, *d*_*z*_ = 0.71, 76% CL), cue-type (*F*(1, 31) = 10.32, *p* < 0.05, *d*_*z*_ = 0.66, 74% CL) and status (*F*(1, 31) = 44.44, *p* < 0.001, *d*_*z*_ = 1.36, 91% CL) were moderated by an interaction between all three factors (*F*(1, 31) = 5.26, *p* < 0.05, *η*_*p*_^*2*^ = 0.15). There was also a cue-type by status interaction (*F*(1, 31) = 10.48, *p* < 0.05, *η*_*p*_^*2*^ = 0.25).

**Table 4:** Experiment 2: Mean reaction times (ms) for correct responses on each task on switch and repeat trials.

	Switch	Repeat
*Episodic task*:		
Old	1540 (364)	1539 (369)
New	1351 (250)	1227 (277)
*Semantic task*:		
Old	1375 (171)	1339 (238)
New	1293 (176)	1271 (224)

A separate analysis for the episodic task revealed main effects of trial-type (*F*(1, 31) = 9.74, *p* < 0.05, *d*_*z*_ = 0.64, 74% CL) and status (*F*(1, 31) = 26.58, *p* < 0.001, *d*_*z*_ = 1.05, 85% CL), which were moderated by an interaction between these two factors (*F*(1, 31) = 5.87, *p* < 0.05, *η*_*p*_^*2*^ = 0.16). This interaction reflects slower responses for switch than for repeat trials for new (*p* < 0.001) but not for old objects. The separate analysis for the semantic task revealed only that responses to new objects were faster than those to old objects (*F*(1, 31) = 18.74, *p* < 0.001, *d*_*z*_ = 0.88, 81% CL).

### ERP analyses

**Fig 5:**
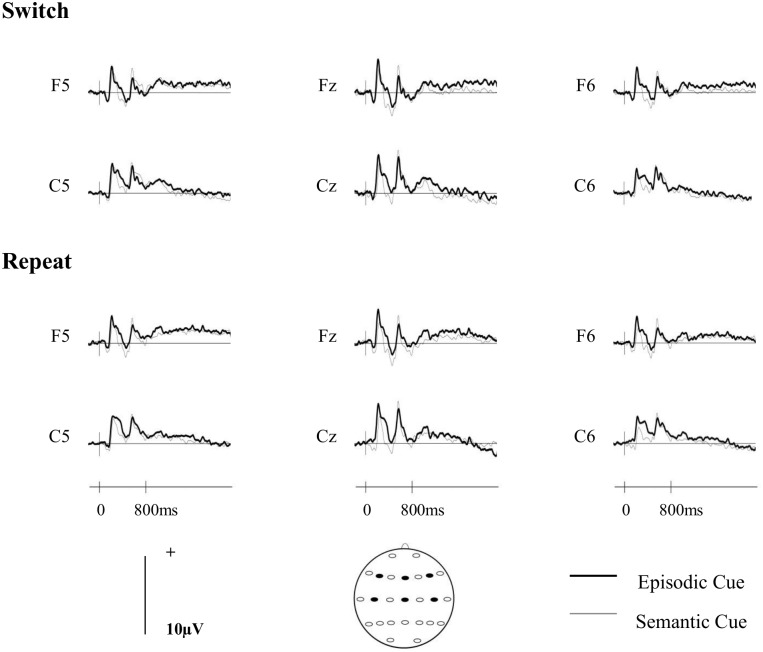
Grand averaged ERPs separated according to cue-type and trial-type. All other details as for Fig 2.

**Fig 6:**
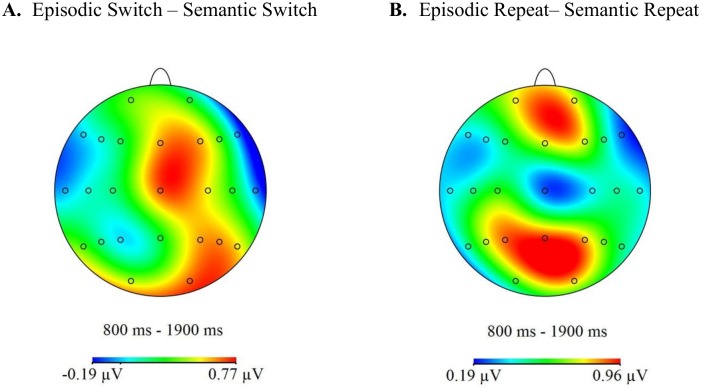
Topographic maps showing the differences between the neural activities associated with the episodic and sematic preparatory cues. All other details as for Fig 3.

As in the first experiment, the ERPs elicited by the two cues indicating which task to complete were analysed over an 800 to 1900ms time window, and the initial analysis included the same 12 sites distributed over fronto-central regions (F3/F4, F5/F6, F7/F8, C3/C4, C5/C6, T7/T8). Fig 5 shows the grand averaged ERP waveforms for each cue- and trial-type at midline, left and right anterior and central sites. Topographic maps depicting the differences between the scalp distributions of the ERPs associated with the two cue- and trial-types are shown in Fig 6. The figures show that while there are some differences on both switch and repeat trials they are small in magnitude.

The initial ANOVA was conducted incorporating the factors of cue-type (episodic/semantic), trial-type (switch/repeat), location in the anterior-posterior plane (anterior/central), hemisphere (left/right), and site (inferior/mid-lateral/superior). This analysis revealed trends only: towards a main effect of cue-type (*F*(1, 31) = 3.03, *p* = 0.092, *d*_*z*_ = 0.31, 62% CL) and an interaction between cue-type and site (*F*(1.4, 44.7) = 3.05, *p* = 0.073, *η*_*p*_^*2*^ = 0.09). Separate exploratory ANOVAs for switch and repeat trials, and anterior and central sites, prompted by the findings in Experiment 1, revealed no reliable effects involving cue-type.

### Bayesian ERP analyses

**Fig 7:**
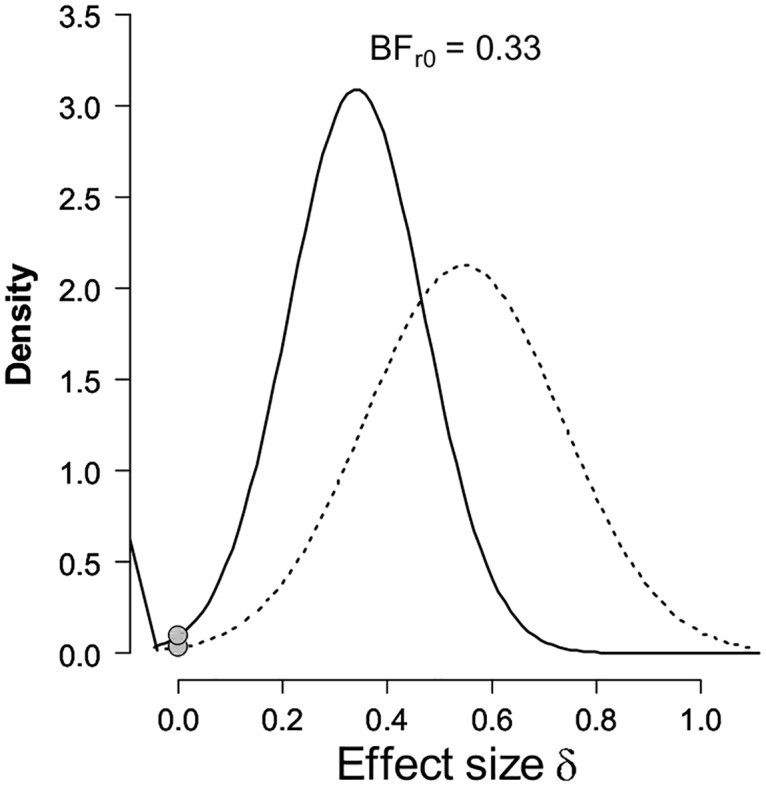
Bayesian results of the replication test for the right-frontal positivity identified previously during preparation for episodic memory retrieval on switch trials [15]. All other information as for Fig 4.

For Experiment 2, as a replication of the divergence identified on switch trials by Evans, Williams [15], at 24 participants there was anecdotal evidence in favour of the null hypothesis (BF = 0.5). The t-value used for this calculation for this experiment with N = 24 was t = 0.92. Guided by the stopping rule, with 32 participants the BF provided substantial evidence in favour of the null (BF = 0.33; Fig 7) [23, 24]. The t-value used for this calculation for this experiment was t = 0.88. These analyses were conducted for the same subset of electrodes and the same time window as for the Bayesian analyses for Experiment 1.

## Discussion

There was no evidence of robust preparatory retrieval processing in Experiment 2 on switch or repeat trials. The findings therefore correspond with those from Experiment 1, in so far as there is, in neither case, evidence for activity at right-frontal scalp sites that has the same direction and time course as that identified in several previous studies and linked to the process of retrieval mode [13–15]. This outcome is again consistent with the view that ERPs do not index a generic signature of retrieval mode, with a somewhat stronger corollary being that there is not a common process engaged during preparation for all kinds of episodic task. The General Discussion below, however, contains a consideration of factors that might render this claim a little premature.

There was also evidence for accuracy switch costs in Experiment 2, and in both experiments evidence that study phase manipulations resulted in levels of response accuracy (for old/new discrimination and location judgments) that were neither at ceiling or floor. Here, they were restricted to old/new discrimination whereas in Experiment 1 they covered old/new discrimination as well as the conditional probabilities of correct source (location) judgments. Lower response accuracies on switch than on repeat trials for source judgments have not been reported uniformly [cf. 13, 14, 26–30]. This is also true for old/new discrimination, although for this measure there are relevant converging data from other paradigms that involve a task-switch of sorts. The ‘revelation effect’ is the name given to the finding that old/new discrimination is sometimes poorer when another kind judgment immediately precedes the old/new decision—for example, solving an anagram of the word to which an old/new judgment is required [31, 32]. The comparison in revelation effect studies is with discrimination in a condition where successive old/new judgments are made without the presence of an intervening task. Given the correspondence between demands in memory switching and revelation effect tasks it is possible that there is a common explanation for the effects that is presumably centred around the consequences of the demands imposed by the preceding task and switching to a different task. If this is a viable suggestion then an important first step will be to assess the changes in performance in task-switching experiments using measures that can provide an indication of changes in bias as well as in old/new discrimination. It has been shown that at least part of the reason for changes in measures of old/new discrimination in the revelation effect is changes in response bias [for discussion and related data points, see: 33–36].

## General Discussion

The most striking element of the findings in both experiments is the absence, on switch and repeat trials, of a modulation with the polarity, temporal and spatial characteristics of that which has been associated previously with the process of retrieval mode. In a number of previous studies divergences between preparatory activity associated with cues to prepare for episodic or semantic retrieval were observed on repeat trials only [10, 12, 13]. This divergence comprised a sustained greater relative positivity associated with cues signalling preparation for episodic rather than for semantic retrieval. A morphologically similar modulation with the same polarity was reported by Evans, Williams [15], but in their experiment the effect was present on switch rather than on repeat trials. Experiment 1 in this report was designed to test the assumption that the switch trial onset of the differences according to cue-type reported by Evans, Williams [15] was a consequence of the fact that, in a departure from previous work, both their tasks required a focus on the same kind of content (spatial information). This has not been the case in previous studies [10, 12, 13]. This design element was also incorporated into Experiment 1 here, with one task requiring semantic knowledge about locations, and other memory for the locations in which items had previously been shown in the task.

If the interpretation offered by Evans, Williams [15] for their observation of a putative index of processes linked to retrieval mode on switch trials is correct, then we should have observed a similar effect in Experiment 1. The failure to replicate the findings of Evans, Williams [15] prompted the execution of Experiment 2. Here the same stimuli as in Experiment 1 were employed but the link between the contents to be focused on in the episodic and semantic tasks was weakened. The design of Experiment 2 thus resembled that used in the majority of previous studies (with respect to the use of episodic and semantic tasks as well as the dissimilarities between contents [12–14, 25]). In light of this, an outcome consistent with that obtained in those studies would have been the presence of a putative index of processes linked to retrieval mode on repeat trials. As noted above, however, this outcome was not observed.

How should these null results be considered? The EEG recording and processing parameters employed here in both experiments are very similar to those used in tasks where putative indices of retrieval mode have been found, suggesting that there is no reason to locate the absence of the effects in the experiments here in data measurement protocols. There is, however, scope to consider other elements of task design that might contribute to the different findings across the experiments reported here and those in other published work.

Two components of task design to which greater attention has been paid in the broad task-switching literature than in memory studies in which task-switches have been employed are: the predictability of the task-sequence [37, 38] and the Response-Cue Interval (RCI)–the time period between the response on a trial and the cue signalling the task to prepare for on the following trial [39]. One possibility is that the absence of a putative index of retrieval mode in the experiments here arose because the task sequence was predictable and participants started to prepare for a switch on repeat trials as soon as they made their response to test items on the preceding trial. If this preparation commenced and was engaged to a reasonable degree in the 1200ms RCI that was employed in these two experiments, then time-locking activity to cue onset on switch trials would reduce markedly the opportunity to observe any such effects.

How does this explanation fare when considering the outcomes of other studies? In some studies in which putative indices of retrieval mode have been observed the task sequence has been unpredictable [12, 14]. In these studies, therefore, there is little incentive to prepare for completion of a particular task in advance of the relevant task-cue. Herron and Wilding [13] had a predictable sequence in so far as switches were required every other trial. The task that was to be switched to was not, however, predictable, because there were three tasks in the experiment, and the order of task completion was determined randomly. This design therefore also should not encourage participants to engage in task-specific preparation before the relevant task cue is encountered. The presence of putative indices of retrieval mode in these experiments is thus consistent with the explanation for the absence of a comparable modulation in the two experiments reported here.

This consistency does not, however, extend to the data reported by Evans, Williams [15]. They used a predictable trial sequence, so the fact that they observed a putative index of retrieval mode on switch trials suggests that an explanation that appeals only to trial sequence cannot accommodate all extant data points. In their experiment, however, the RCI was 500ms—markedly shorter than the 1200ms RCI used in the two experiments reported here. It is possible that this short interval is of insufficient length to enable task-specific preparation to get underway. If this is correct, then indices of preparation might still be observed when activity is time-locked to the following cue.

This explanation covers the majority of outcomes reported here and in prior work. There are, however, other possibilities that cannot be ruled out. One of note is the possibility that we would have observed divergences according to preparatory cue-type had we extended our trial sequences such that there were three or more trials before a switch occurred. Our design precludes testing this possibility, but irrespective of the accuracy of this or the preceding explanation for our data, there are three important corollaries. First, the absence of a putative index of retrieval mode in these two experiments does not licence claims about the existence or otherwise of retrieval mode and its generality. Second, this explanation makes several assumptions about the time over which cognitive sets are adopted, which require testing in appropriately designed follow-up studies. Third, both accounts provide practical pointers towards the task parameters to be employed if retrieval mode and its sequelae are to be investigated: one needs to have a measure of the effect of interest in order to subject it to further questions about how human memory operates and how memory operations are affected by the opportunity to prepare to make memory decisions.

The first of these corollaries—that our null results preclude claims about the presence or otherwise of retrieval mode—applies of course only to the ERP data. The behavioural outcomes in both experiments show performance decrements on switch relative to repeat trials, and can be interpreted as consequences of having adopted a relevant task set only partially on switch trials relative to repeat trials. The behavioural outcomes therefore offer to speak to the important question of the functional significance of adopting retrieval mode: it confers benefits on the accuracy of memory judgments (see the Discussion for Experiment 2). An important next step is to investigate whether, using appropriate experiment parameters, preparatory neural activity varies in a way that predicts changes in behaviour.
